# A multi-method framework for establishing an angular acceleration reference in sensor calibration and uncertainty quantification

**DOI:** 10.1038/s44172-025-00384-8

**Published:** 2025-04-07

**Authors:** Maximilian Gießler, Bernd Waltersberger, Thomas Götz, Robert Rockenfeller

**Affiliations:** 1 Department of Mechanical and Process Engineering, Offenburg University of Applied Sciences, Offenburg, Germany; 2 Mathematical Institute, University of Koblenz, Koblenz, Germany; 3 MTI Mittelrhein, University of Koblenz, Koblenz, Germany

**Keywords:** Computational science, Electrical and electronic engineering, Mechanical engineering

## Abstract

Robots are increasingly being used across various sectors, from industry and healthcare to household applications. In practice, a pivotal challenge is the reaction to unexpected external disturbances, whose real-time feedback often relies on (noisy) sensor measurements. Subsequent inverse-dynamics calculations demand noise-amplifying numerical differentiation, leading to impracticable results. Although much effort has been spent on establishing *direct* measurement approaches, their measurement uncertainty quantification has not or yet insufficiently been tackled in the literature. Here, we propose a multi-method framework to develop an angular acceleration reference and provide evidence that it can serve as a measurement standard to calibrate various kinematic sensors. Within the framework, we use Monte-Carlo simulations to quantify the uncertainty of a direct measurement sensor recently developed by our team; the inertial measurement cluster (IMC). For angular accelerations up to 21 rad/s^2^, the standard deviation of the IMC was on average only 0.3 rad/s^2^ (95% CI: [0.28,0.31]  rad/s^2^), which constitutes a reliable data-sheet record. Further, using least-squares optimization, we show that the deviation of IMC with respect to the reference was not only less on the level of angular acceleration but also on the level of angular velocity and angle, when compared to other direct and indirect measurement methods.

## Abstract (web summary)

Maximilian Gießler and colleagues present a multi-method framework to provide an angular acceleration reference, which significantly improves the accuracy compared to other approaches. The framework could serve as a measurement standard to calibrate various sensors.

## Introduction

Enhancing robust bipedal locomotion in the presence of external perturbations^1^, estimating inertia parameters of body segments in robotic systems^2^, and accurately assessing the dynamics of collaborative manipulators to predict applied external forces^3–5^ are – among others – currently relevant research topics in robotics. Predominantly, these problems are tackled using inverse-dynamics approaches^2–4,6^, for which time-varying joint velocity or position vectors are numerically differentiated once or twice, respectively, to yield joint accelerations and thus forces. It has been shown^3,4^ that obtained joint accelerations are highly sensitive with respect to noise in the kinematic signal, even for state-of-the-art methods in, e.g., inertial parameter estimation^2^. Even worse, numerical differentiation is well-known to amplify even little noise in the data^7^, despite substantial efforts to develop advanced post-processing techniques (e.g., predictive low-pass filter) and state observer techniques (e.g., Kalman filter)^8^.

In robotics as well as in human movement studies, there exist mainly three sources of kinematic data. First, encoders, are typically integrated in actuators to track the joint angle around the one-dimensional joint’s rotation axis. The main limitation is the requirement of onefold or twofold numerical differentiation to track the joint velocity or acceleration, respectively. Second, motion capture (MOCAP) systems, utilizing multiple cameras to track the position of (reflective) markers on the subject of interest^9–12^. Obvious disadvantages of this method are the requirement of a laboratory environment and the need for twofold numerical differentiation. Third, for evaluations outside the laboratory, wearable sensors in the form of inertial measurement units (IMUs) play a central role^13,14^. IMUs enable direct measurements of angular velocity and linear acceleration vectors for various research objectives^15–19^. However, these signals also require numerical differentiation to obtain the angular acceleration vector. Hence, the question arises, if no direct measurement approaches of angular acceleration existed, which would not require numerical differentiation?

Indeed, several direct measurement approaches have been proposed^20–23^. An implicit assumption within these models is the relative orthogonality of the sensors’ position vectors. Given the finite volume of the micro-electromechanical systems (MEMS) used, the unknown exact point of measurement, and possible misalignment in the manufacturing process of the accelerometer, this assumption might be prone to errors. In our previous work^24^, we proposed a more general approach for direct measurement, called “inertial measurement cluster” (IMC), for which the orthogonality assumption was dropped. We applied the IMC to automatically detect balance-recovery responses after external perturbation and qualitatively compared the deviations to previous approaches^20,21^.

Another limitation in the literature is the missing quantitative analysis of measurement accuracies in real-world applications, due to the lack of an angular acceleration reference, see, e.g., ^21,23,25^. Martin and colleagues used an experimental setup of a torsional pendulum equipped with sensors (one degree of freedom) to establish comparative rotational kinematics using the theoretical equation of motion of pendulum motion^26^. The mathematical model described a harmonic pendulum’s motion subjected to viscous friction. The main limitations of this approach were the disregard of both common experimental imperfections such as an eccentric center of mass of the pendulum disk, as well as its tilted rotational axis with respect to the gravitational field. Consequently, their results showed a non-negligible deviation between the pendulum’s computed angular acceleration compared to the potentiometer’s direct measurement^26^, Fig. 6.

In this work, we aim at the validation and uncertainty assessment of the previously presented IMC, while addressing all of the above mentioned shortcomings. Therefore, we set up an angular acceleration reference by utilizing a pendulum oscillating in the gravitational field. The kinematics of the pendulum were measured using a MOCAP system on a set of reflective markers on the pendulum’s rod (positions), as well as IMU and IMC measurements of the angular velocity and acceleration, respectively. A mathematical model of damped oscillation was derived and complete sets of parameters were estimated based on a least-squares deviation from the MOCAP data. Thereby, concurrent global sensitivity analysis^27^ was carried out to obtain reliable initial parameter guesses. All three measurement techniques, including post-processing methods, were compared across all kinematic levels. Subsequently, the parameter uncertainties from the optimization procedure were determined and fed into a Monte-Carlo model simulation, from which the model uncertainties could be estimated. These uncertainties were finally used to reliably assess the IMC noise magnitude.

## Results

To facilitate the understanding of the framework, its underlying simulations and the experimental results, we first provide a concise overview of the full methodology.

### Methods Overview

**Fig. 1:**
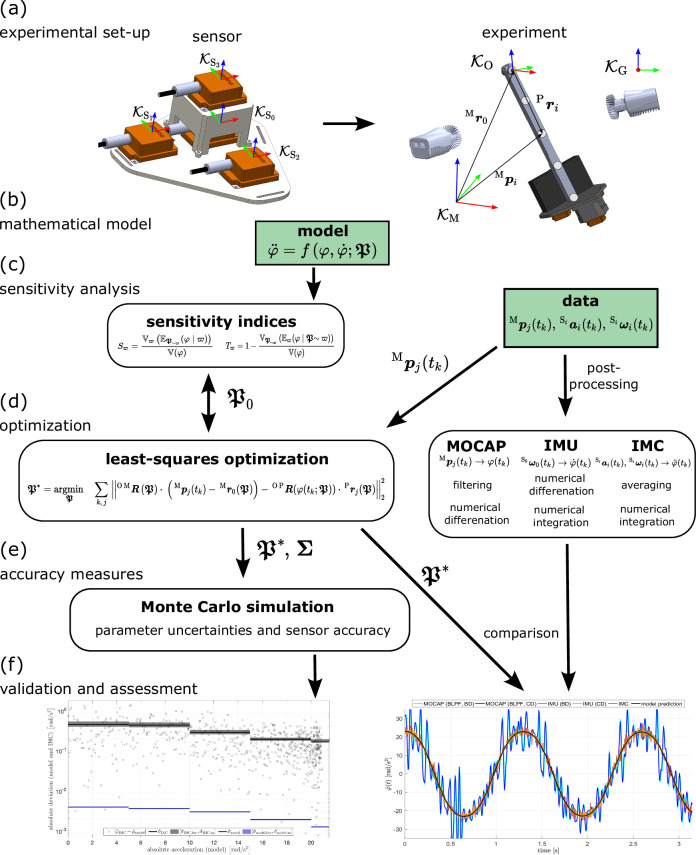
Workflow of the proposed multi-method framework. **a** In the experimental setup, the sensor cluster (IMC) is mounted on a pendulum and its motion is monitored by motion capture (MOCAP), classical IMU, and IMC data. **b** A mathematical model of the pendulum is derived, calculating kinematics $(\varphi ,\dot{\varphi },\ddot{\varphi })$ in dependence of model parameters ${{\boldsymbol{\mathfrak{P}}}}$. **c** The model undergoes a global sensitivity analysis, also comprising variability of the kinematic data. **d** A least-squares optimization is used to determine an optimal data set ${{\boldsymbol{\mathfrak{P}}}}^{* }$, minimizing the distance between model and MOCAP data. Initial parameters and boundary values are determined in reciprocity with the sensitivity analysis. **e** For accuracy measures, a Monte-Carlo simulation reveals both the parameter uncertainties encoded in the covariance matrix ${{\boldsymbol{\Sigma }}}$ and the acceleration-dependent sensor accuracy. **f** The results from the methodological workflow are validated and assessed by comparing the output from the different kinematic data available. For a brief overview, see the wrap-up section. For a detailed explanation, see the Method section.

A variety of methods were employed to (i) establish a reliable reference for determining angular accelerations, (ii) assess our sensor’s measurement uncertainty, and (iii) compare its kinematic measurements to state-of-the-art solutions using MOCAP for angles and IMUs for angular velocities. The underlying concepts are briefly mentioned and displayed in Fig. 1; details can be found in the Method section below.

First, the acceleration sensor was mounted on a pendulum with one rotational degree of freedom (DOF), equipped with four reflective markers M_1_ to M_4_. Kinematics were simultaneously measured by the four internal IMUs (linear acceleration, angular velocities), the IMC (angular acceleration), and 12 infrared cameras (marker position vectors). To make the measurements comparable, a series of transformations between reference frames, post-processing, averaging, filtering, numerical differentiation, and integration had to be conducted. Second, a mathematical model for the damped oscillation ($\ddot{\varphi }=f(\varphi ,\dot{\varphi };{{\boldsymbol{\mathfrak{P}}}})$) was derived, relating the kinematics to a vector comprising 27 parameters ${{\boldsymbol{\mathfrak{P}}}}$. These parameters were physical properties of the pendulum (inverse of the squared gyradius ${R}_{g}^{-2}$, vector to the center of mass ^P^***r***_s_, vectors to the four markers ^P^***r***_1_, …, ^P^***r***_4_, friction coefficient and radius of the bearing *μ* and *r*_b_, the orientation of the pendulum with respect to the gravitational field *θ*, *ψ*, as well as the gravitational constant *g*), initial conditions (*φ*_0_ and ${\dot{\varphi }}_{0}$), and geometrical properties of the MOCAP system (offset ***r***_0_ and orientation *β*, *γ*) Third, a sensitivity analysis of the model equation with respect to the pendulum’s parameters was performed to estimate both local and global effects, measured by their respective sensitivity indices. These indices were utilized to derive a reliable initial parameter guess ${{\boldsymbol{\mathfrak{P}}}}_{0}$. A high parameter sensitivity indicates on the one side that this parameter is important to determine with high accuracy as small changes will lead to significantly different model output, and on the other side that this parameter is likely to be reliably estimated by an optimization procedure. Fourth, a least-squares optimization was performed, resulting in an optimal parameter vector ${{\boldsymbol{\mathfrak{P}}}}^{* }$, minimizing the distance between the MOCAP-measured marker positions ^M^***p***_1_(*t*)…^M^***p***_4_(*t*) and the model output. The initial parameter vector ${{\boldsymbol{\mathfrak{P}}}}_{0}$ and bounds were determined in interaction with the sensitivity analysis results. Fifth, based on the parameter covariances ***Σ*** from the optimization procedure, a Monte-Carlo simulation was carried out to quantify the uncertainty in the modeled acceleration. Uncertainties in the model parameters were thereby translated into variances in model output and compared to measured variances of the IMC. An aim of this analysis was to provide a data sheet with accelerations-dependent sensor accuracy. Last, the measured kinematics were compared with respect to the model output across all three kinematic levels (*φ*, $\dot{\varphi }$, and $\ddot{\varphi }$) and all measurement devices (MOCAP, IMU, IMC), including filtering and post-processing steps.

### Simulation and experimental results

As outlined in the Method section and Supplementary Note 2, the sensitivity of Eqn. (9) with respect to ${\boldsymbol{\mathfrak{P}}}$ was combined with the results of the parameter optimization procedure (Eqn. (11)) to obtain a reliable initial guess ${{\boldsymbol{\mathfrak{P}}}}_{0}$. The resulting optimal parameter vector ${{\boldsymbol{\mathfrak{P}}}}^{* }$ for both experiments E1, E2 (see Methods) are displayed in Table 1 alongside with their 95% confidence intervals (CIs). In particular, experiments E1 and E2 differed in their initial angle and angular velocity. For E1, the initial conditions were *φ*_0_ = − 1.0234 rad and ${\dot{\varphi }}_{0}=0.0053\,\,{\mbox{rad/s}}$, while for E2 they were *φ*_0_ = − 0.4386 rad and ${\dot{\varphi }}_{0}=0.0298\,\,{\mbox{rad/s}}$. The rest of the experimental setup remained unchanged in both cases. Despite having different initial conditions ${\varphi }_{0},{\dot{\varphi }}_{0}$, all remaining parameters were found at comparable values for both experiments. The only exception hereof was the friction coefficient *μ*, which was found at two significantly different values of 0.06 and 0.02, respectively. Further, physical parameters (${R}_{g}^{-2},\,{r}_{{s}_{y}},\,{r}_{{s}_{z}}{,}^{{{{\rm{P}}}}}{{{{\boldsymbol{r}}}}}_{j}$, and *g*) did not substantially deviate from their initial guesses.

**Table 1:** Optimized parameter values

exp.	${R}_{g}^{-2}\,[{{{{\rm{m}}}}}^{-2}]$	${r}_{{{{{\rm{s}}}}}_{y}}\,[{{{\rm{m}}}}]$	${r}_{{{{{\rm{s}}}}}_{z}}\,[{{{\rm{m}}}}]$	*r*_b_ [m]	*μ* [ ]	*φ*_0_ [rad]	${\dot{\varphi }}_{0}\,[{{{\rm{rad}}}}/{{{\rm{s}}}}]$	*θ* [rad]	*ψ* [rad]	*g* [m/s^2^]	*β* [rad]	*γ* [rad]
E1	7.881	-6.048e-5	0.339	9.461e-3	6.198e-2	-1.024	5.176e-3	1.377e-3	1.752e-4	9.809	5.614e-3	0.011
	± 9.282e-3	± 8.811e-8	± 5.965e-4	± 1.037e-5	± 9.531e-5	± 1.360e-4	± 7.696e-6	± 3.053e-6	± 2.49e-7	± 0.019	± 1.819e-4	± 8.197e-4
E2	7.881	-6.592e-5	0.339	9.661e-3	1.910e-2	-0.4329	3.036e-2	1.406e-3	1.998e-4	9.809	6.663e-3	0.011
	± 5.523e-2	± 2.528e-7	± 2.489e-3	± 7.571e-5	± 9.121e-5	± 4.954e-4	± 0.0001905	± 5.89e-6	± 1.177e-6	± 0.045	± 8.044e-4	± 8.758e-3

exp.	^M^*r*_0_ [m]	^P^*r*_1_ [m]	^P^*r*_2_ [m]	^P^*r*_3_ [m]	^P^*r*_4_ [m]
E1	$$\left(\begin{array}{c}0.909\\ -1.401\\ 1.467\end{array}\right)\pm \left(\begin{array}{c}5.923\,{{e-4}}\,\\ 4.259\,{{e-5}}\,\\ 3.562\,{{e-5}}\,\end{array}\right)$$	$$\left(\begin{array}{c}3.151\,{{e-4}}\,\\ -5.164\,{{e-4}}\,\\ -0.081\end{array}\right)\pm \left(\begin{array}{c}0.019\\ 4.054\,{{e-5}}\,\\ 5.765\,{{e-5}}\,\end{array}\right)$$	$$\left(\begin{array}{c}5.049\,{{e-4}}\,\\ -2.459\,{{e-4}}\,\\ -0.181\end{array}\right)\pm \left(\begin{array}{c}6.123\,{{e-4}}\,\\ 3.995\,{{e-5}}\,\\ 6.231\,{{e-5}}\,\end{array}\right)$$	$$\left(\begin{array}{c}5.132\,{{e-4}}\,\\ 5.602\,{{e-5}}\,\\ -0.281\end{array}\right)\pm \left(\begin{array}{c}6.279\,{{e-4}}\,\\ 3.955\,{{e-5}}\,\\ 9.114\,{{e-5}}\,\end{array}\right)$$	$$\left(\begin{array}{c}7.761\,{{e-4}}\,\\ -5.581\,{{e-4}}\,\\ -0.381\end{array}\right)\pm \left(\begin{array}{c}6.433\,{{e-4}}\,\\ 3.983\,{{e-5}}\,\\ 7.123\,{{e-5}}\,\end{array}\right)$$
E2	$$\left(\begin{array}{c}0.909\\ -1.401\\ 1.466\end{array}\right)\pm \left(\begin{array}{c}1.993\,{{e-3}}\,\\ 1.603\,{{e-4}}\,\\ 2.874\,{{e-4}}\,\end{array}\right)$$	$$\left(\begin{array}{c}6.225\,{{e-4}}\,\\ -1.413\,{{e-4}}\,\\ -0.08\end{array}\right)\pm \left(\begin{array}{c}0.045\\ 1.632\,{{e-4}}\,\\ 3.302\,{{e-4}}\,\end{array}\right)$$	$$\left(\begin{array}{c}8.362\,{{e-4}}\,\\ -1.976\,{{e-5}}\,\\ -0.18\end{array}\right)\pm \left(\begin{array}{c}2.571\,{{e-3}}\,\\ 1.589\,{{e-4}}\,\\ 2.870\,{{e-4}}\,\end{array}\right)$$	$$\left(\begin{array}{c}8.137\,{{e-4}}\,\\ 2.922\,{{e-4}}\,\\ -0.281\end{array}\right)\pm \left(\begin{array}{c}3.170\,{{e-3}}\,\\ 1.602\,{{e-4}}\,\\ 3.278\,{{e-4}}\,\end{array}\right)$$	$$\left(\begin{array}{c}1.050\,{{e-3}}\,\\ -4.005\,{{e-4}}\,\\ -0.38\end{array}\right)\pm \left(\begin{array}{c}3.913\,{{e-3}}\,\\ 1.656\,{{e-4}}\,\\ 2.752\,{{e-4}}\,\end{array}\right)$$

Optimal parameter vector ${{\boldsymbol{\mathfrak{P}}}}^{* }$ was calculated from the least-squares procedure, see Fig. 1d or Eqn. (11). The pendulum model with all physical and geometrical parameters was thereby fitted to the experimental data E1 (larger initial pendulum displacement *φ*_0_) and E2 (smaller initial pendulum displacement) using a Levenberg-Marquart algorithm. Point estimates — with units in brackets — are given with the radius of the corresponding 95% CI.

**Fig. 2:**
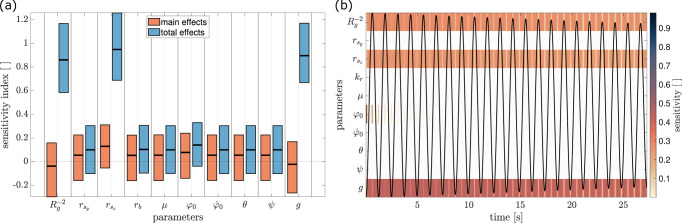
Sensitivity analysis of the pendulum with respect to its model parameters. **a** Local (blue) and global (orange) sensitivity indices around parameter vector ${{\boldsymbol{\mathfrak{P}}}}^{* }$ (experiment E2) with respect to the pendulum model (Fig. 1b and Eqn. (9)). The pendulum parameters are the inverse of the squared gyradius ${R}_{g}^{-2}$, the coordinates of the vector to the center of mass ${r}_{{{{{\rm{s}}}}}_{y}}$ and ${r}_{{{{{\rm{s}}}}}_{z}}$, radius of the bearing and friction coefficient *r*_b_ and *μ*, the initial conditions *φ*_0_ and ${\dot{\varphi }}_{0}$, the orientation of the pendulum with respect to the gravitational field *θ*, *ψ*, as well as the gravitational constant *g*. Black horizontal lines indicate the point estimate while colored areas indicate the 95% CI. The larger the index, the more sensitive the model is to changes in the corresponding parameter. The inverse of the squared gyradius ${R}_{g}^{-2}$, the pendulum’s COM coordinate ${r}_{{{{{\rm{s}}}}}_{z}}$, and the gravitational constant *g* were the most globally influential parameters. **b** This was also verified in **b**, where time-dependent sensitivity of the same model parameters are shown as obtained by the FAST procedure. A typical time-displacement curve is shown as inlay to relate the time courses of the sensitivity values, indicated by the alongside colorbar.

Results from the sensitivity analysis are summarized in Fig. 2. The main (*S*_*i*_) and total sensitivity indices (*T*_*i*_) for the model parameters ${{\mathfrak{P}}}_{i}$ are shown in Fig. 2a together with their corresponding 95% CI. The only parameter, whose main effects were significantly different from zero was the *z*-coordinate of the pendulum’s center of mass (COM) vector ${r}_{{{{{\rm{s}}}}}_{z}}$. The largest total effects were detected for ${R}_{g}^{-2},{r}_{{{{{\rm{s}}}}}_{z}},$ and *g*. As explained in the Method section, particular care was taken to ensure a reliable initial guess of these three parameters. A consistent finding is provided by the FAST analysis (Fig. 2b), for which these three parameters showed the highest sensitivity, if the model was evaluated for a longer time horizon. The light ‘stripes’ in between the otherwise unicolored strips indicate time instances of lower sensitivity, e.g., instances at which $\dot{\varphi }(t)=0$. An additional parameter, *φ*_0_, showed some substantial sensitivity at the beginning of the simulation, which is not surprising as at *t* = 0 it holds that *φ*(*t*) = *φ*_0_ and thus the system is entirely defined by the initial guess.

**Fig. 3:**
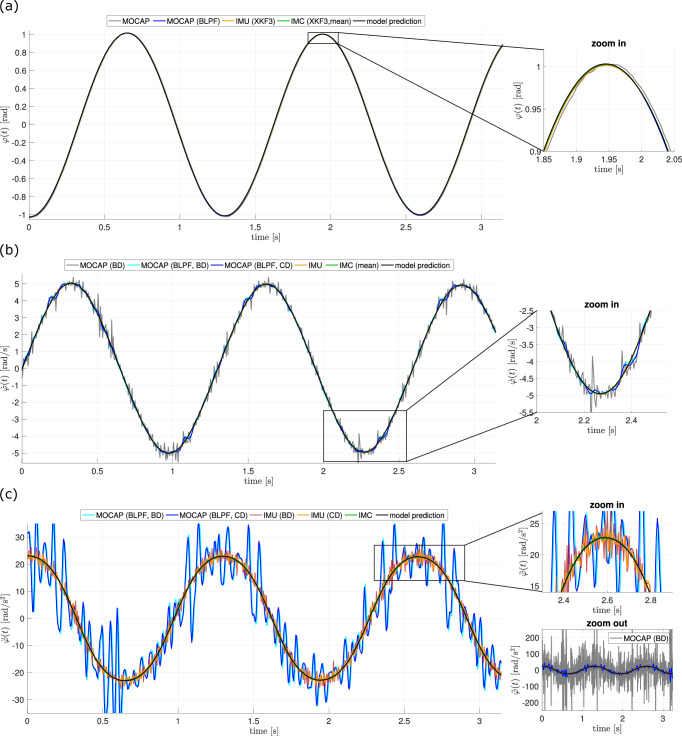
Comparison of measured kinematics with respect to the model output. **a**, **b**, **c** Time courses are shown of (**a**) angles *φ*, (**b**) angular velocities $\dot{\varphi }$, and (**c**) angular accelerations $\ddot{\varphi }$ as obtained from model equation (3) (black lines) and from various measurement methods (colored lines) in experiment E2. Blueish lines correspond to motion capture (MOCAP) measurements where filtering (BLPF), central differentiation (CD), and backward differentiation (BD) are indicated in parentheses. Reddish lines correspond to inertial measurement unit (IMU) measurements with filtering (XKF3) and differentiation methods as well as green lines correspond to inertial measurement cluster (IMC) measurements with filtering (XKF3) and mean calculation likewise indicated. Side inlays show zoomed-in areas to better distinguish the individual lines. Zoomed-out area inlay in **c** illustrates the effect of twice differentiating MOCAP data without filtering. Abbreviations and corresponding residuals are elaborated in Table 2.

The juxtaposition and comparison of the three measurement sources (MOCAP, IMU, IMC) across all kinematic levels ($\varphi (t),\dot{\varphi }(t),\ddot{\varphi }(t)$) and for different error measures (*L*^1^, *L*^2^, *L*^*∞*^) is presented in Fig. 3 for graphical comparison and Table 2 for quantitative error measures. Strikingly, the angle-time curves (Fig. 3a) are graphically equivalent for the raw MOCAP data, the low-pass filtered (BLPF) MOCAP data, the Kalman-filtered IMU data (XKF3), and the IMC data as the mean value of all four IMUs. Residual-wise, MOCAP(BLPF) and IMU(XKF3) performed indeed similar compared to the model equation with the optimized parameter vector, while raw MOCAP data showed three to four times increased residuals, and averaging for the IMC reduced the residuum by 55–57%. For the angular velocity-time courses, the disadvantage of the numerical differentiation procedures (BD and CD) became manifest. Compared to the IMU as a reference, the MOCAP residuals were 8-49 times larger, depending on the measure and differentiation method. Even for filtered MOCAP data, the residuals were 3.4 to 7.5 times larger. The averaged IMC on the other hand showed again an improvement in residuals by 54–57%. In the angular acceleration-time regime, the reference was given by the IMC, with respect to which the MOCAP showed an approximately 30-fold (filtered) or a  > 300-fold (unfiltered) increased residuum, respectively. The single IMU, whose data had to be numerically differentiated, showed residuals increased by a factor 2.2-7.2. In all cases, the centrally differentiated data were closer to the model output than the backwardly differentiated data. This effect vanished for the filtered MOCAP data.

**Table 2:** Comparison of error measures

kinematics	method	error measure		
		*L*^1^	*L*^2^	*L*^*∞*^
*φ*(*t*) [rad]	reference values factors for	0.0038	0.0048	0.0097
	⋅ MOCAP	4.16	3.74	3.03
	⋅ MOCAP(BLPF)	**1**	**1**	**1**
	⋅ IMU(XKF3)	1.04	0.97	0.89
	⋅ IMC(XKF3, mean)	0.45	0.43	0.45
$\dot{\varphi }(t)\quad \left[\,{\mbox{rad/s}}\,\right]$	reference values factors for	0.0192	0.0218	0.0097
	⋅ MOCAP (BD)	10.89	14.45	49.23
	⋅ MOCAP (CD)	7.95	9.83	27.95
	⋅ MOCAP (BLPF, BD)	3.88	4.58	7.54
	⋅ MOCAP (BLPF, CD)	3.4	4.24	7.33
	⋅ IMU	**1**	**1**	**1**
	⋅ IMC(mean)	0.43	0.44	0.46
$\ddot{\varphi }(t)\quad \left[{{{\rm{rad}}}}/{{{{\rm{s}}}}}^{2}\right]$	reference values factors for	0.2159	0.297	1.3811
	⋅ MOCAP (BD)	311.55	326.12	355.11
	⋅ MOCAP (CD)	311.63	326.12	355.03
	⋅ MOCAP (BLPF, BD)	29.03	29.16	35.3
	⋅ MOCAP (BLPF, CD)	29.03	29.13	35.23
	⋅ IMU (BD)	7.18	6.46	4.53
	⋅ IMU (CD)	3.67	3.28	2.2
	⋅ IMC	**1**	**1**	**1**

Error measures (*L*^1^, *L*^2^, *L*^*∞*^) of kinematic data ($\varphi (t),\dot{\varphi }(t),\ddot{\varphi }(t)$) were obtained from different sources (MOCAP, IMU, IMC) in experiment E2. Reference values of the residuals are given together with their corresponding prefactors, where **1** is used to indicate the reference measurements. All deviations are given with respect to the model (Eqn. (3)) using the optimized parameter vector ${{\boldsymbol{\mathfrak{P}}}}^{* }$. For the non-reference measurements, the method of post-processing is given in parentheses, i.e. Butterworth low-pass filtering (BLPF), quaternion-based Kalman filtering (XKF3), central differentiation (CD), backward differentiation (BD), and averaging (mean).

**Fig. 4:**
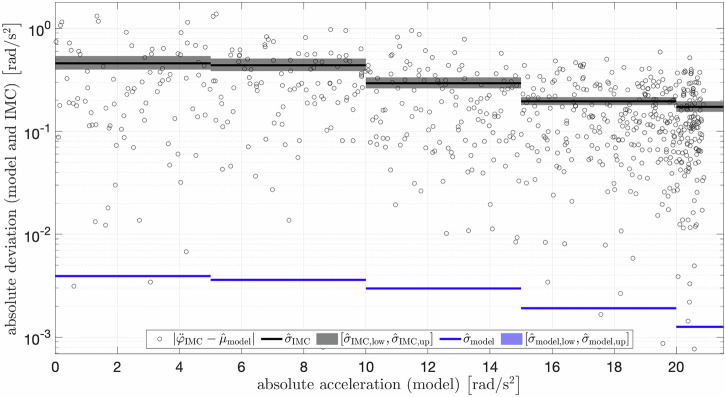
Uncertainty quantification in the modeled and measured angular acceleration. Deviations of the inertial measurement cluster (IMC) measurements (${\ddot{\varphi }}_{{{{\rm{IMC}}}}}$) and the mean of the Monte-Carlo-sampled model outputs (${\hat{\mu }}_{{{{\rm{model}}}}}$) — measured by their absolute difference $| {\ddot{\varphi }}_{{{{\rm{IMC}}}}}-{\hat{\mu }}_{{{{\rm{model}}}}}|$ (black circles) — are plotted versus the absolute value of ${\hat{\mu }}_{{{{\rm{model}}}}}$. Respective estimators of standard deviations for the model (${\hat{\sigma }}_{{{{\rm{model}}}}}$, blue lines) and the IMC measurements ${\hat{\sigma }}_{{{{\rm{IMC}}}}}$ (black lines) are shown together with their confidence intervals (${\hat{\sigma }}_{{{{\rm{IMC,low}}}}}$, ${\hat{\sigma }}_{{{{\rm{IMC}}}},{{{\rm{up}}}}}$, ${\hat{\sigma }}_{{{{\rm{model,low}}}}}$, and ${\hat{\sigma }}_{{{{\rm{model,up}}}}}$; defined by lower and upper bounds, shaded areas around lines). Acceleration values were grouped into intervals of 5 rad/s. For clarity, only the absolute deviations on a logarithmic scale are shown.

To assess the uncertainty of the IMC acceleration measurements in the light of parameter uncertainty, the deviation of *M* = 100,000 sampled model evaluations to both the IMC measurements (${\ddot{\varphi }}_{{{{\rm{IMC}}}}}({t}_{k})$) and the mean of the Monte-Carlo-sampled model outputs (${\hat{\mu }}_{{{{\rm{model}}}}}({t}_{k})$) are depicted in Fig. 4. Therefore, estimators for the standard deviations ${\hat{\sigma }}_{{{{\rm{IMC}}}}}({t}_{k})$ and ${\hat{\sigma }}_{{{{\rm{model}}}}}({t}_{k})$ were calculated within bins of 5 rad/s^2^ together with their 95% CIs. With increasing angular acceleration, the uncertainty of the IMC tendentially decreased; from ${\hat{\sigma }}_{{{{\rm{IMC}}}}}=0.458\,{{\mbox{rad/s}}}^{2}$ (95% CI: [0.398, 0.539]  rad/s^2^) between 0-5 rad/s^2^ to 0.173 rad/s^2^ (95% CI: [0.155, 0.196] rad/s^2^) at above 20 rad/s^2^. The radius of the 95% CIs in each case lay between 20% and 30% of the nominal value and were not affected by the sample size, as only the deviation of the *N*_bin_ data points to ${\hat{\mu }}_{{{{\rm{model}}}}}$ within each bin were considered. The same tendency but two magnitudes lower held for the uncertainty in the model outputs given randomly sampled parameters; from ${\hat{\sigma }}_{{{{\rm{model}}}}}=0.00392\,{{\mbox{rad/s}}}^{2}$ between 0-5 rad/s^2^ to 0.00126 rad/s^2^ at above 20 rad/s^2^. The radius of the 95% CIs in each model case lay below 0.1% of the nominal value due to the high sample size. Resultant, the CI for ${\hat{\sigma }}_{{{{\rm{res}}}}}$ approximately split into thirds from ${\hat{\sigma }}_{{{{\rm{res}}}}}=0.458\,{{\mbox{rad/s}}}^{2}$ (95% CI: [0.399, 0.539] rad/s^2^) at 0-5 rad/s to ${\hat{\sigma }}_{{{{\rm{res}}}}}=0.173\,{{\mbox{rad/s}}}^{2}$ (95% CI: [0.155, 0.196] rad/s^2^) at  > 20 rad/s. If no bins were considered, the average deviation accounted for ${\hat{\sigma }}_{{{{\rm{res}}}}}=0.297\,{{\mbox{rad/s}}}^{2}$ (95% CI: [0.282, 0.313] rad/s^2^). The confidence intervals for the potential bias ${\hat{b}}_{{{{\rm{IMC}}}}}$ were altogether contained in the interval [ − 0.1, 0.1] rad/s^2^ each containing zero, with the absolute largest value of ${\hat{b}}_{{{{\rm{IMC}}}}}$ being less than 0.015 rad/s^2^.

## Discussion

To extract body segments’ kinematics in robotics, different sensors exist that can measure directly the segments’ joint positions (encoder or potentiometers), or the absolute angular velocity ***ω*** and the linear acceleration ***a*** vectors (IMU). Utilizing such sensors for inverse dynamics evaluations inevitably requires noise amplifying numerical differentiation to assess the angular acceleration vector $\dot{{{{\boldsymbol{\omega }}}}}$, which leads to impracticable results^2–6^. Alternative approaches for indirect or direct angular acceleration measurement are not or insufficiently validated regarding their measurement uncertainty^21,23,25,26^. In recent studies^7,24^, we presented, validated, and assessed a direct measurement method for the angular accelerations of moving objects: the inertial measurement cluster (IMC). The validation was based on computational modeling that provided a reference curve, combined with a qualitative comparison using experimental data. Despite extensive efforts to model both stochastic and deterministic errors of the IMC^24^, uncertainty persists regarding the transferability of the computational model’s results to real-word applications. But there is a lack of evidence as to whether these modeled errors comprehensively capture the IMC’s measurement uncertainty and, consequently, reflect the real-world behavior. To address these concerns, we developed a pendulum experiment, which was redundantly evaluated using both a theoretical model and multiple measurement methods across kinematic levels. The descriptive parameters of the mathematical model were fitted to accurately map the directly measured position vector trajectories (MOCAP), enabling the mathematical model to serve as an angular acceleration reference. An a-priori global sensitivity analysis supported the optimization process, while an a-posteriori Monte-Carlo simulation enabled the uncertainty quantification of the model and the IMC.

### Angular acceleration reference

Although the mathematical model of a swinging pendulum (Eqn. (3)) seems simple enough, the final formulation of the resulting EOM (Eqn. (9)) contains some crucial assumptions. In particular, we placed importance on including the relative positioning and orientation of the pendulum with respect to the kinematic measurement systems and the gravitational field — a problem known to have been ignored by Martin et al.^26^. Next, we acknowledged the a-priori unknown mass distribution of the pendulum’s final design, since inertia parameters of cables, measurement devices, as well as mounting imperfection effects, cannot be accurately predicted. Regarding physical friction effects, it is well known that these cannot be mapped arbitrarily accurately with macroscopic models. Therefore, we chose a ball bearing concept for which we assumed that the dissipation can be sufficiently modeled by a simple Coulomb friction model. In pilot tests, we additionally evaluated other friction models, such as viscous friction (proportional to $\dot{\varphi }(t;{\boldsymbol{\mathfrak{P}}})$) or the combination of viscous and Coulomb friction. The pilot test results indicated that the combination of viscous and Coulomb friction yielded physically non-meaningful optimized parameter values (e.g., a negative value for *μ*). In contrast, modeling purely Coulomb friction resulted in a physically meaningful friction coefficient *μ* and the smallest deviation to the measured markers’ position vector trajectories ***p***_*j*_(*t*_*k*_). The effect of sticking was neglected, as it obviously did not occur during the experiments.

Additionally, careful consideration was taken to estimate the initial parameter set ${{\boldsymbol{\mathfrak{P}}}}_{0}$ for the least-square optimization. CAD-derived geometries, high-precision scales, bearing data sheets, and an online tool of the Physical-Technical Federal Institute served this purpose, see the Method section for details. Further, as many modeling papers were found to have flaws in assessing their parameter sensitivities^28,29^, we exploited local, global, as well as time-dependent sensitivity measures^27,30^. Only three parameters (${R}_{{{{\rm{g}}}}}^{-2},{r}_{{{{{\rm{s}}}}}_{z}},g$) were found to have significant (CIs not containing zero) global sensitivities, even over the whole time period. It was also shown that the local measure alone would not have revealed that insight.

Regarding the validity, results obtained from the mathematical model evaluated with the fitted model parameters ${{\boldsymbol{\mathfrak{P}}}}^{* }$ showed a sufficiently precise mapping of the markers’ position vector trajectories ***p***_*j*_(*t*_*k*_) (RMSE_E1_ = 0.00061 m and RMSE_E2_ = 0.0014 m). As additional evidence, we propose the following findings. First, the optimized values ${{\boldsymbol{\mathfrak{P}}}}^{* }$ did not substantially sheer off their initial values, and also found similar values for both experiments, indicating that the model was indeed well-posed. The only parameter showing a high volatility was the friction coefficient *μ*, which had higher values when the pendulum was more deflected and thus showed higher angular velocities (*μ*_E1_ = 0.06; *μ*_E2_ = 0.02). Consequently, this indicates that the current applied simple Coulomb friction model fails to account for all dissipative effects, as evidenced by the varying friction coefficient *μ* observed across both experiments. Hence, the friction coefficient itself may depend on the pendulum’s applied bearing load and is angular velocity-dependent, matching the claim of a Stribeck curve^31^. A future refinement of the model could thus be to incorporate a more sophisticated friction model for ball bearings that account for the dependence of *μ* on the bearing load and angular velocity state, as proposed in the literature^32^. However, there is no evidence whether the parameters of such models are valid for the specific angular velocity range and bearing load conditions of the pendulum. Consequently, all parameters of these sophisticated friction models would need to be included in the optimization process, which might introduce overfitting and result in physically non-meaningful optimized parameter values (cf. the pilot study results for the combination of viscous and Coulomb friction). Here, we refrained from this refinement due to the already vanishingly low residuals, the low sensitivity of the model with respect to *μ*, and the fact that such simple models are common practice in the literature^33,34^.

Second, we compared our model’s predicted pendulum angle with the estimation of the post-processed MOCAP(BLPF) angular data, resulting in consistently low RMSEs for both experiments (E1: 0.0048 rad, E2: 0.0059 rad). Third, likewise low RMSEs were found for IMU(XKF3) and IMC(XKF3,mean) data, of which no information was used to drive the parameter optimization procedure. By comparing our results to previously proposed approaches^26^, Fig. 6, Martin and colleagues obtained a significantly greater deviation between the direct position measurement (potentiometer) and the applied mathematical model for the pendulum angle. This highlights the potential of our approach being considerable as a reference.

As a second step, we quantified the measurement uncertainty of the proposed model (Fig. 4). Therefore, we used the methodology of the Monte-Carlo simulation to resolve the impact of the uncertainty regarding the statistically interdependent model parameters ${\boldsymbol{\mathfrak{P}}}$ on $\ddot{\varphi }(t;{\boldsymbol{\mathfrak{P}}})$ (cf. International Organization for Standardization^35^). Herein, we postulated that parameters of ${\boldsymbol{\mathfrak{P}}}$ are normally distributed and, consequently, we modeled their statistical dependence by a multivariate normal distribution. The obtained uncertainty of the model – on the level of accurately determining the angular acceleration – was assessed and likewise found to be acceleration-dependent; it split into thirds from ${\hat{\sigma }}_{{{{\rm{model}}}}}=0.0039\,{{\mbox{rad/s}}}^{2}$ at low velocities (0-5 rad/s) to ${\hat{\sigma }}_{{{{\rm{model}}}}}=0.0013\,{{\mbox{rad/s}}}^{2}$ at high velocities ( > 20 rad/s). Note again that these values were given with a confidence region of width less than 0.1% of their nominal value. In the literature, information about uncertainties for reference measurements on angular acceleration is missing, and hence no comparison of our values is possible. As the sample size of the underlying Monte-Carlo simulation was chosen large enough to ensure a narrow estimate of the standard deviation, these values can be considered reliable. In conclusion, the proposed reference can be considered reliable and simultaneously can serve as a measurement standard.

At the same time, our approach also provides reference curves $\varphi (t;{{\boldsymbol{\mathfrak{P}}}}^{* })$ for the angle and $\dot{\varphi }(t;{{\boldsymbol{\mathfrak{P}}}}^{* })$ for the angular velocity. Through the relative kinematic relationship (Eqn. (1)), for example, the linear acceleration vector of any material point of the pendulum can be determined based on the *φ*, $\dot{\varphi }$, and $\ddot{\varphi }$. This allows the quantification of measurement uncertainty as well as the comparison of various sensors for the kinematic quantities relevant to multi-body dynamics. Moreover, this approach represents an adequate alternative to the quantification of measurement uncertainty for, e.g., MEMS sensors compared to established approaches using the gravitational field for linear acceleration or precise turntables for the angular velocity vector^36,37^.

### Measurement uncertainty of IMC

Our main goal was to quantify the uncertainty of our angular acceleration measurement sensor, IMC, in order to provide a reliable data-sheet record. For the quantification of the measurement uncertainty of the IMC also the measurement uncertainty of the model must be taken into account. Therefore, the deviation of the IMC measurements (${\ddot{\varphi }}_{{{{\rm{IMC}}}}}$) from the Monte-Carlo-determined mean (${\hat{\mu }}_{{{{\rm{model}}}}}$), i.e. ${\hat{\sigma }}_{{{{\rm{IMC}}}}}$, as well as the uncertainty of the model itself (${\hat{\sigma }}_{{{{\rm{model}}}}}$) had to be determined.

Regarding the limitations of this approach, we noted that the unknown bias of the IMC to the reference is inevitably contained within ${\hat{\sigma }}_{{{{\rm{IMC}}}}}^{2}$. Yet, we assumed the empirical mean ${\hat{\mu }}_{{{{\rm{model}}}}}({t}_{k})$ to be sufficiently close to the expected value of $\ddot{\varphi }$. A strong argument in favor of this assumption is given by the fact that the maximum deviation between ${\hat{\mu }}_{{{{\rm{model}}}}}(t)$ obtained from the Monte-Carlo simulation and $\ddot{\varphi }(t;{{\boldsymbol{\mathfrak{P}}}}^{* })$ obtained from the least-squares optimization was less than 0.001 rad/s^2^. Further, dividing the entire measurement range into equal-sized bins, we showed that the estimated mean deviation (bias) ${\hat{b}}_{{{{\rm{IMC}}}}}$ within each bin did in no case significantly differ from zero. Here, we used bins (bin width of 5 rad/s^2^) in order to obtain a sufficiently large sample of measurements. It is important to note that this assumes constant variance and bias within each bin.

Our findings showed that the IMC uncertainty mainly depended on the deviation between IMC measurements and model output, as the model uncertainty was two magnitudes smaller. The uncertainty was acceleration-dependent and on average accounted for ${\hat{\sigma }}_{{{{\rm{res}}}}}=0.297\,{{\mbox{rad/s}}}^{2}$ (95% CI: [0.282, 0.313] rad/s^2^). Note that we were able to provide upper and lower bounds for the deviation within each bin, which exceeds the information typically found in sensor data-sheets. Therein, typically one value for the standard deviation depending on the sampling frequency is given that approximately holds for the entire measurement range.

As stated above, regarding the objective comparison of the IMC to other indirect measurement methods of the angular acceleration with respect to the proposed reference $\ddot{\varphi }(t;{{\boldsymbol{\mathfrak{P}}}}^{* })$, we cannot provide any statement on the behavior of twice differentiated angle measurements by encoders typically used in robotics, as in the experimental setup there were neither encoders nor actuators installed. Although MOCAP measurements have little relevance in robotics, they are a standard tool in human motion analysis^9,10^ and hence served here as a reference of direct position measurements. In any case, the assumption that plain or post-processed twice numerically differentiated encoder measurement data do not provide meaningful results was already confirmed with data from the literature^2–6^. It is further underpinned by the known error propagation of backward difference methods, which amplify noise depending on the sample frequency. Indeed, the comparison of the deviation of the measurement data with respect to the reference showed that the IMC average error was approximately 3.3 times and 6.5 times smaller than evaluated by the central difference or the real-time capable backward difference quotient of single IMUs. To qualitatively indicate the impact of twice numerical differentiation we can consider MOCAP-derived accelerations. The errors were 29 or 326 times larger with or without using filters, respectively, yielding results unusable for further analyses. This clearly highlights the key advantage of direct measurement with respect to the precise extraction of angular acceleration vectors. Without using post-processing, the once numerically differentiated angular velocity, directly measured by a precise gyroscope, represents the most reliable indirect measurement method.

On the level of angular velocities, $\dot{\varphi }(t;{{\boldsymbol{\mathfrak{P}}}}^{* })$, the IMC showed an improvement by a factor 2 compared to the direct measurements by the IMU. This factor is not surprising, since averaging all four gyroscopes of the IMC provides a quadrupled sample size, which scales the error by a factor $1/\sqrt{4}=1/2$ according to the Bienaymé’s identity for pairwise independent random values. The residuals of the once numerically differentiated MOCAP data were 4.2 to 14 times higher than compared to the single IMU, again highlighting its relevance in kinematic measurements.

For the comparison with respect to the reference angular output $\varphi (t;{{\boldsymbol{\mathfrak{P}}}}^{* })$, the data obtained via the sensor fusion algorithm (XKF3) from the single IMUs was equally good, and the residuals of the IMC were even better, again by a factor 2, compared to the post-processed and filtered MOCAP angular predictions. However, we want to clearly point out that, this might be realistic for the here applied measurement duration of about 20 s to 25 s. During this short period, the inevitable drift caused by the underlying numerical integration (solving a quaternion differential equation system for orientation estimation) was not significantly noticeable. On the other hand, for a longer measurement duration, the direct position measurement of the MOCAP or the angle measurement of encoders have a key advantage, as both principles are not affected by a drift due to numerical integration. Nevertheless, the existing redundancy of the angular velocity measurement offers new possibilities through specially adapted sensor fusion algorithms (similar to those proposed in^24^) to achieve further accuracy improvements for the angular velocity vector measurement or to reduce the impact of drift for orientation estimation. As a last note, the consistency of deviations between sensor measurements and fitted model with respect to different error measures (*L*^1^, *L*^2^, and *L*^*∞*^) across all kinematic levels serves as a further indicator of the reference model quality.

Considering the limitations of the multi-method framework, the achievable amplitude of $\ddot{\varphi }$ of the swinging pendulum and thus also of $\dot{\varphi }$ for the pendulum oscillating in the gravitational field is bounded by its mass and mass distribution (***I***^(0)^ and ***r***_s_). The measurement uncertainty quantification is directly linked and therefore limited to the pendulum’s $\ddot{\varphi }$ amplitude. Due to the observed decreasing measurement uncertainty regarding higher angular acceleration ranges, we assume that the proposed pendulum covers the ranges with the highest measurement uncertainty. Concerning the current geometrical size and weight of the IMC, it might affect noticeably the dynamics of the body segment. The miniaturization is currently in progress and especially the multi-method framework plays a crucial role in providing an accurate reference for future small and light-weight IMCs.

## Methods

### Nomenclature

**Fig. 5:**
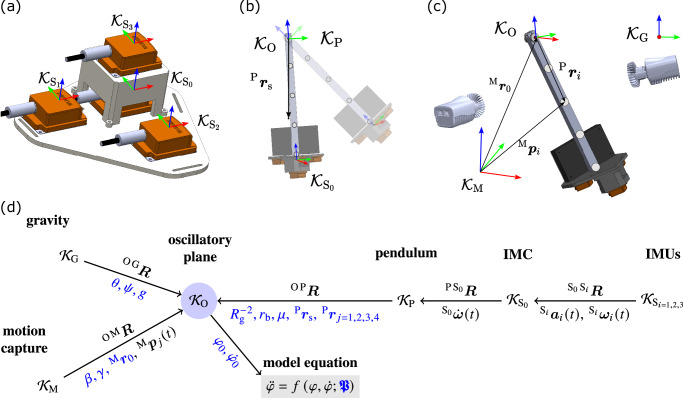
Schematics of the pendulum setup and reference frames. **a**, **b**, **c** The panels show an overview of the chain of reference frames belonging to (**a**) the whole IMC sensor (${{{{\mathcal{K}}}}}_{{{{{\rm{S}}}}}_{0}}$) and the single IMUs (${{{{\mathcal{K}}}}}_{{{{{\rm{S}}}}}_{i}}$), (**b**) the plane of oscillation (${{{{\mathcal{K}}}}}_{{{{\rm{O}}}}}$) and the body-fixed pendulum (${{{{\mathcal{K}}}}}_{{{{\rm{P}}}}}$), (**c)** the motion capture system (${{{{\mathcal{K}}}}}_{{{{\rm{M}}}}}$) and the gravitational field (${{{{\mathcal{K}}}}}_{{{{\rm{G}}}}}$). **d** Transformation between each two reference frames ${{{{\mathcal{K}}}}}_{{{{\rm{A}}}}}$ and ${{{{\mathcal{K}}}}}_{{{{\rm{B}}}}}$ is conducted via a corresponding rotation matrix ^B A^***R***, given above the arrows. Below the arrows, all measurements are printed in black, all parameters ${\boldsymbol{\mathfrak{P}}}$ in blue. The measurements are the position vectors of the four reflective markers ^M^***p***_*j*=1,…,4_(*t*) pointing from the motion capture system’s origin, the angular acceleration vector ${\scriptstyle{{{{\rm{S}}}}}_{0}\atop}\!\dot{{{{\boldsymbol{\omega }}}}}(t)$ measured by the inertial measurement cluster (IMC), and the linear acceleration and angular velocity vectors ${\scriptstyle{{{{\rm{S}}}}}_{i}\atop}\!{{{{\boldsymbol{a}}}}}_{i}(t)$ and ${\scriptstyle{{{{\rm{S}}}}}_{i}\atop}\!{{{{\boldsymbol{\omega }}}}}_{i}(t)$ measured by the inertial measurement units (IMU). The parameters are the physical properties of the pendulum (inverse of the squared gyradius ${R}_{g}^{-2}$, vector to the center of mass ^P^***r***_s_, vectors to the four markers ^P^***r***_*j*=1,2,3,4_, radius of the bearing and friction coefficient *r*_b_ and *μ*, the orientation of the pendulum with respect to the gravitational field *θ*, *ψ*, as well as the gravitational constant *g*), initial conditions (*φ*_0_ and ${\dot{\varphi }}_{0}$), and geometrical properties of the motion capture system (vector pointing from the origin to the pendulum’s pivot 0 ^M^***r***_0_ and orientation *β*, *γ*). The model equation is eventually solved in the reference frame of the oscillatory plane, to which all measurements and orientations are transformed.

A boldface symbol ***v***^(*P*)^ refers to a vector or a second order tensor, in general, if needed, with a reference point *P* denoted by an upper right index in brackets. We stipulate that without any further reference to a coordinate frame, the symbol is to be understood as the 3 × 1 or 3 × 3 coordinate matrix of the vector or tensor, respectively, with respect to the inertial frame that represents the oscillatory plane ${{{{\mathcal{K}}}}}_{{{{\rm{O}}}}}$, cf. Fig. 5. Any vector has its origin in the origin of ${{{{\mathcal{K}}}}}_{{{{\rm{O}}}}}$. A further additional upper left index as in ^F^***v***^(*P*)^ is used to indicate the coordinate matrix of the vector or tensor with respect to frame ${{{{\mathcal{K}}}}}_{{{{\rm{F}}}}}$, in case it differs from ${{{{\mathcal{K}}}}}_{{{{\rm{O}}}}}$.

Another convention used herein concerns the dependency of mathematical objects on the parameter vector ${\boldsymbol{\mathfrak{P}}}$. In cases where we want to stress this dependency, concrete parameters or the whole vector are added in parentheses, e.g., ^O G^***R***(*θ*, *ψ*) for the rotation matrix between frames ${{{{\mathcal{K}}}}}_{{{{\rm{G}}}}}$ and ${{{{\mathcal{K}}}}}_{{{{\rm{O}}}}}$ depending on the Cardan angles *θ* and *ψ*, or ${{{{\boldsymbol{r}}}}}_{{{{\rm{0}}}}}({\boldsymbol{\mathfrak{P}}})$ for the displacement vector of ${{{{\mathcal{K}}}}}_{{{{\rm{O}}}}}$ with respect to ${{{{\mathcal{K}}}}}_{{{{\rm{M}}}}}$, or $\varphi (t;{\boldsymbol{\mathfrak{P}}})$ for the model equation solution.

### Experimental setup

To obtain data that were sufficiently dynamical for non-trivial measurements, while simultaneously being easy enough to model, two variations of a simple pendulum experiment were carried out and their kinematics captured.

The pendulum itself consisted of a sufficiently stiff aluminum rod, so that it can be assumed to behave rigidly, equipped with four reflective markers mounted in pre-drilled boreholes, see Fig. 5c. Two hybrid ball bearings (HC 628 TN) were used to realize the bearing concept. The experiments themselves consisted of deflections of the rod by hand, once by a smaller angle ( ≈ 25°) and once by a larger angle ( ≈ 58°). During the pendulum motion, a twelve-camera (infrared Miqus M3) motion capture system (Qualisys AB, Gothenburg, Sweden) recorded the coordinates of the markers’ position vectors at a sampling frequency of 200 Hz. At the end of the rod, an IMU-based sensor with a sampling frequency of likewise 200 Hz measured the pendulum’s kinematics as explained in the following subsection. Data were taken after the first full oscillation to ensure the system was transient. Each measurement had a duration of approximately 25 s, leading to *N*_E1_ = 4402 sample points for experiment E1 and *N*_E2_ = 4458 for E2.

### Sensor measuring principle

In Gießler et al.^24^, we have introduced and validated the so-called *inertial measurement cluster* (IMC), i.e. a sensor directly measuring angular acceleration. Its built-up and measurement principle can be briefly summarized as follows: A total of four IMUs of type Xsens MTi-20 VRU (Movella Inc., Nevada, USA) — each consisting of a 3D gyroscope plus 3D accelerometer — were mounted on a custom base plate as shown in Fig. 5a. An arbitrary IMU, denoted S_0_, served as a reference, while the other three IMUs (S_*i*_ with *i* = 1, 2, 3) were placed with linearly independent connecting vectors ***r***_*i*,0_ with respect to the reference. The linear independence of these three vectors and of their respective covectors can be exploited to calculate the angular acceleration $\dot{{{{\boldsymbol{\omega }}}}}$ from the linear acceleration ***a***_*i*_ and the angular velocity ***ω*** extracted as the mean value of the angular velocities measured by the four IMUs *i* = 0, . . , 3, respectively. Concretely, as shown in Gießler et al.^24^, Eqn. (1)-(7), the well-known rigid body equation

1 $${{{{\boldsymbol{a}}}}}_{i}={{{{\boldsymbol{a}}}}}_{0}+\dot{{{{\boldsymbol{\omega }}}}}\times {{{{\boldsymbol{r}}}}}_{i,0}+{{{\boldsymbol{\omega }}}}\times ({{{\boldsymbol{\omega }}}}\times {{{{\boldsymbol{r}}}}}_{i,0})$$

can be solved for $\dot{{{{\boldsymbol{\omega }}}}}$ in the dual vector space and eventually writes

2 $$\dot{{{{\boldsymbol{\omega }}}}}={\left(\begin{array}{c}{\left({{{{\boldsymbol{r}}}}}_{1,0}\times {{{{\boldsymbol{r}}}}}_{2,0}\right)}^{T}\\ {\left({{{{\boldsymbol{r}}}}}_{2,0}\times {{{{\boldsymbol{r}}}}}_{3,0}\right)}^{T}\\ {\left({{{{\boldsymbol{r}}}}}_{3,0}\times {{{{\boldsymbol{r}}}}}_{1,0}\right)}^{T}\end{array}\right)}^{-1}\cdot \left(\begin{array}{c}\langle {{{{\boldsymbol{a}}}}}_{1}-{{{{\boldsymbol{a}}}}}_{0}-{{{\boldsymbol{\omega }}}}\times ({{{\boldsymbol{\omega }}}}\times {{{{\boldsymbol{r}}}}}_{1,0}),{{{{\boldsymbol{r}}}}}_{2,0}\rangle \\ \langle {{{{\boldsymbol{a}}}}}_{2}-{{{{\boldsymbol{a}}}}}_{0}-{{{\boldsymbol{\omega }}}}\times ({{{\boldsymbol{\omega }}}}\times {{{{\boldsymbol{r}}}}}_{2,0}),{{{{\boldsymbol{r}}}}}_{3,0}\rangle \\ \langle {{{{\boldsymbol{a}}}}}_{3}-{{{{\boldsymbol{a}}}}}_{0}-{{{\boldsymbol{\omega }}}}\times ({{{\boldsymbol{\omega }}}}\times {{{{\boldsymbol{r}}}}}_{3,0}),{{{{\boldsymbol{r}}}}}_{1,0}\rangle \end{array}\right),$$

where 〈 ,  〉 denotes the standard scalar product. Note that the inverse matrix in Eqn. (2) always exists due to the linear independency of the vectors ***r***_*i*,0_.

### Model built-up: reference frames and parameters

The aim of this section is to motivate the model, which was used as a reference to assess the obtained kinematic measurements. A planar damped oscillation can be described by a second order differential equation of the form

3 $$\ddot{\varphi }=f\left(\varphi ,\dot{\varphi };{\boldsymbol{\mathfrak{P}}}\right),\qquad \varphi (0)={\varphi }_{0},\,\,\dot{\varphi }(0)={\dot{\varphi }}_{0},$$

where *φ* = *φ*(*t*) denotes the time evolution of the deflection angle of the pendulum with respect to its resting state within its oscillatory plane, $\dot{\varphi }$ and $\ddot{\varphi }$ the first and second time derivative, respectively, ${\boldsymbol{\mathfrak{P}}}\in {{\mathbb{R}}}^{n}$ the vector of model parameters, and $f:\,{\mathbb{R}}\times {\mathbb{R}}\times {{\mathbb{R}}}^{n}\to {\mathbb{R}}$ a suitable right side.

In our experimental set-up, data output with respect to different reference frames had to be combined, as shown in Fig. 5. First, this concerned the linear accelerations ${\scriptstyle{{{{\rm{S}}}}}_{i}\atop}\!{{{{\boldsymbol{a}}}}}_{i}(t)$ and angular velocities ${\scriptstyle{{{{\rm{S}}}}}_{i}\atop}\!{{{{\boldsymbol{\omega }}}}}_{i}(t)$, for *i* = 0, 1, 2, 3, from each of the four IMUs, provided in their own reference frames ${{{{\mathcal{K}}}}}_{{{{{\rm{S}}}}}_{i}}$ These outputs were combined within the reference frame of the reference IMU, i.e. ${{{{\mathcal{K}}}}}_{{{{{\rm{S}}}}}_{0}}$, to yield the angular acceleration ${\scriptstyle{{{{\rm{S}}}}}_{0}\atop}\!\dot{{{{\boldsymbol{\omega }}}}}(t)$ of the IMC. Second, the pendulum itself was assigned a body-fixed reference frame ${{{{\mathcal{K}}}}}_{{{{\rm{P}}}}}$, which moved in the reference frame of the oscillatory plane ${{{{\mathcal{K}}}}}_{{{{\rm{O}}}}}$. Third, the positions of the four reflective markers (M_*j*_ with *j* = 1, …, 4) were gathered by the motion capture system in its reference frame ${{{{\mathcal{K}}}}}_{{{{\rm{M}}}}}$ and hence denoted ^M^***p***_*j*_(*t*) to distinguish them from the coordinates of the markers (i.e. the parameters) in ${{{{\mathcal{K}}}}}_{{{{\rm{P}}}}}$, where they write ^P^***r***_*j*_. Finally, the experiment took place in the gravitational field coordinate system ${{{{\mathcal{K}}}}}_{{{{\rm{G}}}}}$.

In order to write down Eqn. (3) as simple as possible, all quantities were transformed in the reference frame ${{{{\mathcal{K}}}}}_{{{{\rm{O}}}}}$ of the oscillatory *y*-*z*-plane. While the issue of the relative orientations of the IMUs was addressed in the preceding subsection, several additional assumptions were required for the remaining reference frames. To transform the line of gravity (*z*-axis) in ${{{{\mathcal{K}}}}}_{{{{\rm{G}}}}}$ into ${{{{\mathcal{K}}}}}_{{{{\rm{O}}}}}$, two rotations *θ* and *ψ* around the *x*- and *y*-axis, respectively, were conducted. The corresponding rotation matrix is written as ^O G^***R*** = ^O G^***R***(*θ*, *ψ*), cf. Woernle^38^ and Fig. 5d. A similar transformation was conducted for the motion capture frame ${{{{\mathcal{K}}}}}_{{{{\rm{M}}}}}$, which was additionally translated by a vector ^M^***r***_0_ to match the origin of ${{{{\mathcal{K}}}}}_{{{{\rm{O}}}}}$ and rotated around the *y*- and *z*-axis by angles *β* and *γ*, respectively, to match the *x*-axes, i.e. ^O M^***R*** = ^O M^***R***(*β*, *γ*). The pendulum reference frame, ${{{{\mathcal{K}}}}}_{{{{\rm{P}}}}}$, was also chosen to match origin and *x*-axis with ${{{{\mathcal{K}}}}}_{{{{\rm{O}}}}}$ such that the transformation between these two frames can be described by an elemental rotation ${}^{{{{\rm{O}}}}{{{\rm{P}}}}}{{{\boldsymbol{R}}}}{ = }^{{{{\rm{O}}}}{{{\rm{P}}}}}{{{\boldsymbol{R}}}}(\varphi (t;{\boldsymbol{\mathfrak{P}}}))$. It is worth noting that the coordinates of the reflective markers within ${{{{\mathcal{K}}}}}_{{{{\rm{P}}}}}$ were assumed constant over time.

To formulate model equation (3) within ${{{{\mathcal{K}}}}}_{{{{\rm{O}}}}}$, we start with the general equations of motion (EOM)

4 $$m{{{{\boldsymbol{a}}}}}_{{{{\rm{s}}}}}=m{{{\boldsymbol{g}}}}+{{{{\boldsymbol{F}}}}}_{{{{\rm{r}}}}}+{{{{\boldsymbol{F}}}}}_{{{{\rm{f}}}}}$$

5 $${{{{\boldsymbol{I}}}}}^{(0)}\dot{{{{\boldsymbol{\omega }}}}}+{{{\boldsymbol{\omega }}}}\times {{{{\boldsymbol{I}}}}}^{(0)}{{{\boldsymbol{\omega }}}}={{{{\boldsymbol{r}}}}}_{{{{\rm{s}}}}}\times m{{{\boldsymbol{g}}}}+{{{{\boldsymbol{M}}}}}_{{{{\rm{f}}}}}^{(0)},$$

where *m* denotes the mass of the pendulum, ***a***_s_ the linear acceleration of its COM, ***g*** the gravity vector, ***F***_r_ the reaction force, ***F***_f_ the friction force, ***I***^(0)^ the inertia tensor with respect to the pendulum’s pivot 0 (the origin of ${{{{\mathcal{K}}}}}_{{{{\rm{O}}}}}$), ***r***_s_ the vector pointing from 0 to the pendulum’s COM, and ${{{{\boldsymbol{M}}}}}_{{{{\rm{f}}}}}^{(0)}$ the frictional moment with respect to 0. For the frictional force and the resulting frictional moment of the bearing, ***F***_f_ and ${{{{\boldsymbol{M}}}}}_{{{{\rm{f}}}}}^{(0)}$, we assume both to be proportional to ***F***_r_ and thus ∣∣***F***_f_∣∣ = *μ*∣∣***F***_r_∣∣ as well as ${{{{\boldsymbol{M}}}}}_{{{{\rm{f}}}}}^{(0)}=\mu {r}_{{{{\rm{b}}}}}| | {{{{\boldsymbol{F}}}}}_{{{{\rm{r}}}}}| | {{{{\boldsymbol{e}}}}}_{{{{{\rm{O}}}}}_{x}}$, with the friction coefficient *μ* and its lever arm *r*_b_ (approximately the outer ring radius). The effective line of ***F***_f_ is assumed to be orthogonal to ***F***_r_ and the oscillation axis ${{{{\boldsymbol{e}}}}}_{{{{{\rm{O}}}}}_{x}}$ of ${{{{\mathcal{K}}}}}_{{{{\rm{O}}}}}$, so that

6 $${{{{\boldsymbol{F}}}}}_{{{{\rm{f}}}}}={{{\rm{sign}}}}(\dot{\varphi })\mu {{{{\boldsymbol{e}}}}}_{{{{{\rm{O}}}}}_{x}}\times {{{{\boldsymbol{F}}}}}_{{{{\rm{r}}}}}$$

applies. Inserting Eqn. (6) into (4) gives

7 $${{{{\boldsymbol{F}}}}}_{{{{\rm{r}}}}}+{{{\rm{sign}}}}(\dot{\varphi })\mu {{{{\boldsymbol{e}}}}}_{{{{{\rm{O}}}}}_{x}}\times {{{{\boldsymbol{F}}}}}_{{{{\rm{r}}}}}=m{{{{\boldsymbol{a}}}}}_{{{{\rm{s}}}}}-m{{{\boldsymbol{g}}}}.$$

Further, due to the positioning of ${{{{\mathcal{K}}}}}_{{{{\rm{O}}}}}$, it holds that ${{{\boldsymbol{\omega }}}}=\dot{\varphi }{{{{\boldsymbol{e}}}}}_{{{{{\rm{O}}}}}_{x}}$, i.e. the angular velocity points towards the pendulum *x*-axis. Solving Eqn. (5) for the *x*-coordinate yields

8 $${I}_{xx}^{(0)}\ddot{\varphi }+{{{\rm{sign}}}}(\dot{\varphi })\langle {{{{\boldsymbol{M}}}}}_{{{{\rm{f}}}}}^{(0)},{{{{\boldsymbol{e}}}}}_{{{{{\rm{O}}}}}_{x}}\rangle +\langle {{{{\boldsymbol{r}}}}}_{{{{\rm{s}}}}}\times m{{{\boldsymbol{g}}}},{{{{\boldsymbol{e}}}}}_{{{{{\rm{O}}}}}_{x}}\rangle =0,$$

which expands in scalar notation to

9 $$\begin{array}{l}\ddot{\varphi }+{{{\rm{sign}}}}(\dot{\varphi }){R}_{{{{\rm{g}}}}}^{-2}\mu {r}_{{{{\rm{b}}}}}\\ {\left(\frac{{({k}_{1}\ddot{\varphi }+{c}_{1}+{c}_{2})}^{2}}{{({\mu }^{2}+1)}^{2}}+\frac{{({k}_{2}\ddot{\varphi }+{c}_{3}+{c}_{4})}^{2}}{{({\mu }^{2}+1)}^{2}}\right)}^{\frac{1}{2}}+{c}_{5}=0.\end{array}$$

More details regarding the terms *c*_1_ to *c*_4_, *k*_1_, and *k*_2_ and Eqn. (9) are shown in Supplementary Note 1. Note three things: First, Eqn. (9) is normalized by replacing the quotient $m/{I}_{xx}^{(0)}$, i.e. the inverse of the squared gyradius of the pendulum around the *x*-axis, with the symbol ${R}_{{{{\rm{g}}}}}^{-2}$. Second, the sign function term ${{{\rm{sign}}}}(\dot{\varphi })$ ought to ensure that friction is always counteracting the motion. We do not explicitly take the case of sticking into account, since during the observed period of the experiment no sticking occurred. Third, although Eqn. (9) is formulated implicitly, it can be analytically solved for $\ddot{\varphi }$ by solving a quadratic equation, yielding explicit Eqn. (3).

Putting together the final parameter vector ${\boldsymbol{\mathfrak{P}}}$, we end up with eight physical parameters that occur in the model equation (${R}_{{{{\rm{g}}}}}^{-2},{r}_{{{{{\rm{s}}}}}_{y}},{r}_{{{{{\rm{s}}}}}_{z}},{r}_{{{{\rm{b}}}}},\mu ,\theta ,\psi ,g$), two initial conditions (${\varphi }_{0},{\dot{\varphi }}_{0}$), and 17 auxiliary, geometrical parameters (${r}_{{0}_{x}},{r}_{{0}_{y}},{r}_{{0}_{z}},\gamma ,\beta ,{r}_{{1}_{x}},{r}_{{1}_{y}},{r}_{{1}_{z}},\ldots ,{r}_{{4}_{x}},{r}_{{4}_{y}},{r}_{{4}_{z}}$) yielding a total of 27 unknowns:

10 $$\begin{array}{rcl}{\boldsymbol{\mathfrak{P}}}&=&\left({R}_{{{{\rm{g}}}}}^{-2},{r}_{{{{{\rm{s}}}}}_{y}},{r}_{{{{{\rm{s}}}}}_{z}},{r}_{{{{\rm{b}}}}},\mu ,\theta ,\psi ,g,{\varphi }_{0},{\dot{\varphi }}_{0},{r}_{{0}_{x}},{r}_{{0}_{y}},{r}_{{0}_{z}},\right.\\ &&{\left.\gamma ,\beta ,{r}_{{1}_{x}},{r}_{{1}_{y}},{r}_{{1}_{z}},\ldots ,{r}_{{4}_{x}},{r}_{{4}_{y}},{r}_{{4}_{z}}\right)}^{T},\end{array}$$

where *g* denotes the absolute value of the gravitational acceleration, ${r}_{{{{{\rm{s}}}}}_{y}}$, ${r}_{{{{{\rm{s}}}}}_{z}}$ are the components of ^P^***r***_s_ in the *y*-*z*-plane, ${r}_{{0}_{x}}$, ${r}_{{0}_{y}}$, and ${r}_{{0}_{z}}$ are the components of ^M^***r***_0_, and ${r}_{{1}_{x}},{r}_{{1}_{y}},{r}_{{1}_{z}},\ldots ,{r}_{{4}_{x}},{r}_{{4}_{y}},{r}_{{4}_{z}}$ are the components of ^P^***r***_*j*=1,2,3,4_.

### Optimization procedure

To estimate the parameter values and quantify their uncertainties, the positional data from the MOCAP system were fitted by the pendulum’s EOM in the ${{{{\mathcal{K}}}}}_{{{{\rm{O}}}}}$ reference frame. The optimal parameter vector ${{\boldsymbol{\mathfrak{P}}}}^{* }$ was obtained using the nlinfit routine – a Levenberg-Marquart algorithm – in MatLab (Version 2023b, Mathworks, Natick, USA), minimizing the sum of squared distances between the MOCAP measurements at time increments *t*_*k*_ and the corresponding model evaluations. The following equation writes the problem for the more general formulation in ${{{{\mathcal{K}}}}}_{{{{\rm{O}}}}}$ (Eqn. (11a)) and for the more concrete formulation (Eqn. (11b)) according to Fig. 5

11a $$\hskip 5pt{{\boldsymbol{\mathfrak{P}}}}^{* } = {{{{\rm{arg}}}}\,{{{\rm{min}}}} }_{{\boldsymbol{\mathfrak{P}}}}{\sum}_{k,\,j}\,{\left| \left| {{{{\boldsymbol{p}}}}}_{j}({t}_{k})-\left({{{{\boldsymbol{r}}}}}_{0}({\boldsymbol{\mathfrak{P}}}) + {{{{\boldsymbol{r}}}}}_{j}({t}_{k};{\boldsymbol{\mathfrak{P}}})\right)\right| \right| }_{2}^{2},$$

11b $$= 	 {{{{\rm{arg}}}}\,{{{\rm{min}}}} }_{{{\boldsymbol{\mathfrak{P}}}}} \sum\limits_{k,\,j}\,\left\| {\scriptstyle{{{{\rm{OM}}}}}\atop}\!{{{\boldsymbol{R}}}}\left({{\boldsymbol{\mathfrak{P}}}}\right)\cdot \left({\scriptstyle{{{{\rm{M}}}}}\atop}\!{{{\boldsymbol{p}}}}_j(t_k) -{\scriptstyle{{{{\rm{M}}}}}\atop}\!{{{\boldsymbol{r}}}}_0({{\boldsymbol{\mathfrak{P}}}})\right) \right. \\ 	\left. - {\scriptstyle{{{{\rm{OP}}}}}\atop}\!{{{\boldsymbol{R}}}}(\varphi(t_k;{{\boldsymbol{\mathfrak{P}}}})) \cdot {\scriptstyle{{{{\rm{P}}}}}\atop}\!{{{\boldsymbol{r}}}}_{j}({{\boldsymbol{\mathfrak{P}}}})\right\|^2_2, $$

where $\varphi (t;{\boldsymbol{\mathfrak{P}}})$ was determined using Eqn. (9). Note that all 27 parameters were optimized simultaneously with no intermediate steps. The determination of the initial values ${{\boldsymbol{\mathfrak{P}}}}_{0}$ for the Levenberg-Marquart algorithm is explained in more detail in Supplementary Note 2.

Although problem Eqn. (11) is formulated as to minimize the squared Euclidean distance, we evaluated other measures as well to assess the deviation of time-dependent data $(\varphi (t),\dot{\varphi }(t),\ddot{\varphi }(t))$ from the model output at *t*_*k*_ and for ${{\boldsymbol{\mathfrak{P}}}}^{* }$. In particular, we evaluated

- the total (Manhattan) distance ${L}^{1}:= \frac{1}{N}{\sum }_{k = 1}^{N} \vert {{{\rm{data}}}}({t}_{k})-{{{\rm{model}}}}({t}_{k};{{\boldsymbol{\mathfrak{P}}}}^{* })\vert ,$
- the normalized Euclidean distance ${L}^{2}:= {\big(\frac{1}{N}{\sum }_{k = 1}^{N}{\vert {{{\rm{data}}}}({t}_{k})-{{{\rm{model}}}}({t}_{k};{{\boldsymbol{\mathfrak{P}}}}^{* })\vert }^{2}\big)}^{1/2}$, as well as
- the maximum (Chebyshev) distance ${L}^{\infty }:= {\max }_{k}\vert \,{{\mbox{data}}}({t}_{k})-{{\mbox{model}}}({t}_{k};{{\boldsymbol{\mathfrak{P}}}}^{* })\vert ,$

where *N* denotes the number of time instances. Note that these distances did not depend on the number of markers and thus did not require a summation over *j*.

### Uncertainty assessment

An advantage of the nlinfit procedure from the prior subsection is that the Jacobian of the optimization problem with respect to all parameters – and thus the covariance matrix ***Σ*** comprising the mean squared error – is obtained by an approximation using finite differences. In a subsequent step, ***Σ*** was used to perform a Monte-Carlo simulation to assess the uncertainty of the modeled acceleration with respect to the uncertainty of the parameters. The methodology was oriented on the ISO/BIPM ‘Guide to the Expression of Uncertainty in Measurement’^35^. A total of *M* = 100,000 samples of the parameter vector ${({\hat{{\boldsymbol{\mathfrak{P}}}}}_{j})}_{j = 1,\ldots ,M}$ were drawn from a multivariate normal distribution with means ${{\boldsymbol{\mathfrak{P}}}}^{* }$ and covariances ***Σ*** using the MatLab routine mvnrnd. Equation (9) was solved numerically for each parameters vector ${\hat{{\boldsymbol{\mathfrak{P}}}}}_{j}$ using MatLab’s differential equation solver ode78, with setting the relative tolerance to 10^−8^ and the absolute tolerance to 10^−9^.

Subsequently, the mean acceleration ${\hat{\mu }}_{{{{\rm{model}}}}}(t)$ of the *M* realizations ${\ddot{\varphi }}_{{{{\rm{model}}}}}(t,{\hat{{\boldsymbol{\mathfrak{P}}}}}_{j})$ was calculated. To provide a reliable data-sheet entry for the IMC and to check for a possibly acceleration-dependent uncertainty, we grouped the mean model accelerations into bins of width 5 rad/s^2^. Within each bin, for every corresponding time instance *t*_*k*_ we calculated the *N*_bin_ differences ${\hat{\mu }}_{{{{\rm{model}}}}}({t}_{k})-{\ddot{\varphi }}_{{{{\rm{IMC}}}}}({t}_{k})$ and the *N*_bin_ ⋅ *M* differences ${\hat{\mu }}_{{{{\rm{model}}}}}({t}_{k})-{\ddot{\varphi }}_{{{{\rm{model}}}}}({t}_{k},{\hat{{\boldsymbol{\mathfrak{P}}}}}_{j})$, where *k* = 1, …, *N*_bin_ and *j* = 1, …, *M*. A normal distribution was fitted through both sets of differences using MatLab’s fitdist routine, yielding estimators for the standard deviations ${\hat{\sigma }}_{{{{\rm{IMC}}}}}=\sqrt{\frac{1}{{N}_{{{{\rm{bin}}}}}-1}\left(\mathop{\sum }_{k = 1}^{{N}_{{{{\rm{bin}}}}}}{({\hat{\mu }}_{{{{\rm{model}}}}}({t}_{k})-{\ddot{\varphi }}_{{{{\rm{IMC}}}}}({t}_{k}))}^{2}\right)}$ and ${\hat{\sigma }}_{{{{\rm{model}}}},k}=\sqrt{\frac{1}{M-1}\left(\mathop{\sum }_{j = 1}^{M}{({\hat{\mu }}_{{{{\rm{model}}}}}({t}_{k})-{\ddot{\varphi }}_{{{{\rm{model}}}}}({t}_{k},{\hat{{\boldsymbol{\mathfrak{P}}}}}_{j}))}^{2}\right)}$, respectively. The 95% CI for the sensor uncertainty, i.e. $[{\hat{\sigma }}_{{{{\rm{IMC,low}}}}},{\hat{\sigma }}_{{{{\rm{IMC}}}},{{{\rm{up}}}}}]$, whose width only depended on *N*_bin_, was likewise provided by the fitdist routine. To obtain an estimator for the standard deviation with respect to all *N*_bin_ sample points, we used a convex combination of ${\hat{\sigma }}_{{{{\rm{model}}}},k}$, i.e. ${\hat{\sigma }}_{{{{\rm{model}}}}}^{2}=\frac{1}{{N}_{{{{\rm{bin}}}}}}\mathop{\sum }_{k = 1}^{{N}_{{{{\rm{bin}}}}}}{\hat{\sigma }}_{{{{\rm{model}}}},k}^{2}$ (cf. refined Satterthwaite method^39^). Likewise, for the CI of the model uncertainty, i.e. $[{\hat{\sigma }}_{{{{\rm{model,low}}}}},{\hat{\sigma }}_{{{{\rm{model,up}}}}}]$, we used a refined Satterthwaite method^39^ to calculate an average CI for each bin, whose width thus only depended on *M*. The value *M* = 100,000 of the Monte-Carlo samples was chosen to ensure a width of the ${\hat{\sigma }}_{{{{\rm{model}}}}}$ CI to be less than 0.1% of the nominal value. Further, the bias of the IMC with respect to the reference is inevitably contained within each bin’s estimator ${\hat{\sigma }}_{{{{\rm{IMC}}}}}^{2}$. Hence, we herein assume the corresponding random variables for the differences ${D}_{{{{\rm{IMC}}}}} \sim {{{\mathcal{N}}}}({b}_{{{{\rm{IMC}}}}},{\sigma }_{{{{\rm{IMC}}}}}^{2})$ and ${D}_{{{{\rm{model}}}}} \sim {{{\mathcal{N}}}}(0,{\sigma }_{{{{\rm{model}}}}}^{2})$ to be normally distributed, independent, and in case of the IMC to contain an unknown bias *b*_IMC_. Hence, the resultant uncertainty ${D}_{{{{\rm{res}}}}}$ of the IMC is a superposition of the deviations, i.e. ${D}_{{{{\rm{res}}}}}={D}_{{{{\rm{IMC}}}}}-{D}_{{{{\rm{model}}}}} \sim {{{\mathcal{N}}}}\left({b}_{{{{\rm{IMC}}}}},{\sigma }_{{{{\rm{res}}}}}^{2}\right)$, whose standard deviation can be estimated by

12 $${\hat{\sigma }}_{{{{\rm{res}}}}}=\sqrt{{\hat{\sigma }}_{{{{\rm{IMC}}}}}^{2}+{\hat{\sigma }}_{{{{\rm{model}}}}}^{2}}.$$

Acknowledging these different numbers of realization underlying ${\hat{\sigma }}_{{{{\rm{IMC}}}}}$ and ${\hat{\sigma }}_{{{{\rm{model}}}}}$, we approximate the resultant 95% CI $[{\hat{\sigma }}_{{{{\rm{res,low}}}}},{\hat{\sigma }}_{{{{\rm{res,up}}}}}]$ again by the refined Satterthwaite method^39^, Eqn. (2.2). The bias’ estimator ${\hat{b}}_{{{{\rm{IMC}}}}}$ and its 95% CI $[{\hat{b}}_{{{{\rm{IMC,low}}}}},{\hat{b}}_{{{{\rm{IMC}}}},{{{\rm{up}}}}}]$ were likewise obtained from the fitdist routine.

### Sensitivity analysis of the pendulum with respect to its model parameters

In order to assess which parameters $\varpi \in {\boldsymbol{\mathfrak{P}}}$ (Eqn. (10)) of the ODE model (Eqn. (9)) have the most prominent effects — and should hence be treated with higher accuracy or caution — a global sensitivity analysis is carried out^27^, whose bases are briefly described here. Therefore, let $\varphi =\varphi (t;{\boldsymbol{\mathfrak{P}}})$ denote the ODE-model solution, obtained for the parameter vector ${\boldsymbol{\mathfrak{P}}}$. The Cartesian product of the 95% CIs derived from the covariance matrix ***Σ*** forms a hyperrectangle ${{{\mathcal{H}}}}$ around ${\boldsymbol{\mathfrak{P}}}$. In this context, for an arbitrary parameter *ϖ*, let ${{\boldsymbol{\mathfrak{P}}}}_{ \sim \varpi }$ indicate that all parameters of ${\boldsymbol{\mathfrak{P}}}$ are allowed to vary within the boundaries of ${{{\mathcal{H}}}}$; except *ϖ* which is being fixed. As shown for Saltelli et al. ^27^, ch. 4, the state *φ* can be decomposed into conditional expected values (${\mathbb{E}}$) and variances (${\mathbb{V}}$) to obtain both a *first order sensitivity (main) index S*_*ϖ*_ as well as a *total sensitivity index T*_*ϖ*_. The former is defined by

13 $${S}_{\varpi }=\frac{{{\mathbb{V}}}_{\varpi }\left({{\mathbb{E}}}_{{{\boldsymbol{\mathfrak{P}}}}_{ \sim \varpi }}(\varphi | \varpi )\right)}{{\mathbb{V}}(\varphi )},$$

representing the effect of solely varying parameter *ϖ*, but simultaneously taking known variations of other parameters into account. Here, the indices on ${\mathbb{E}}$ and ${\mathbb{V}}$ indicate that all possible parameter values in the relevant subspace of ${{{\mathcal{H}}}}$ are considered for the calculation. The total sensitivity index is defined by

14 $${T}_{\varpi }=1-\frac{{{\mathbb{V}}}_{{{\boldsymbol{\mathfrak{P}}}}_{ \sim \varpi }}\left({{\mathbb{E}}}_{\varpi }(\varphi | {{\boldsymbol{\mathfrak{P}}}}_{ \sim \varpi })\right)}{{\mathbb{V}}(\varphi )},$$

representing the effect of varying parameter *ϖ* including interactions of any order with every remaining parameter. In order to efficiently calculate time-dependent sensitivity indices *S*_*i*_(*t*), Fourier amplitude sensitivity testing (FAST) is applied via the “sensitivity analysis for everybody” (SAFE) toolbox^30^.

### Data post-processing

We recall that the data output of our experiments contains (i) raw positional data ^M^***p***_*j*_(*t*) from the MOCAP system, (ii) raw angular velocity data ${\scriptstyle{{{{\rm{S}}}}}_{i}\atop}\!{{{{\boldsymbol{\omega }}}}}_{i}(t)$ from the IMUs, and (iii) calculated (see above) angular acceleration data ${\scriptstyle{{{{\rm{S}}}}}_{0}\atop}\!\dot{{{{\boldsymbol{\omega }}}}}(t)$ from the IMC, see also Fig. 5. Transforming these values to the reference frame ${{{{\mathcal{K}}}}}_{{{{\rm{O}}}}}$, we annotate the corresponding data for *φ*(*t*) and its derivatives as *φ*_M_(*t*) (MOCAP), ${\dot{\varphi }}_{{{{{\rm{S}}}}}_{i}}(t)$ (IMU), and ${\ddot{\varphi }}_{{{{{\rm{S}}}}}_{0}}(t)$ (IMC). To make these measurements comparable to each other, *φ*_M_(*t*) had to be numerically differentiated twice and ${\dot{\varphi }}_{{{{{\rm{S}}}}}_{i}}(t)$ once.

Thereby, we further discriminate between possible applications, e.g., real-time versus a-posteriori data availability. If real-time processing of data is necessary, numerical differentiation can only be conducted using backward-difference (BD) methods, whereas a-posteriori processing allows for exploiting the advantages of central-difference (CD) methods. As an additional a-posteriori tool in order to reduce noise, a 4th order Butterworth low-pass filter (BLPF) was applied to the MOCAP data.

For the opposing comparison, i.e. to calculate angular positions out of the angular velocity measurements ${\ddot{\varphi }}_{{{{{\rm{S}}}}}_{i}}(t)$, the IMU offers a quaternion-based Kalman filtering routine (XKF3^40,41^), which allows for a real-time (200 Hz) numerical integration. To further reduce noise for the IMC-based states ${\varphi }_{{{{{\rm{S}}}}}_{0}}(t)$ and ${\dot{\varphi }}_{{{{{\rm{S}}}}}_{0}}(t)$, the arithmetic mean of all four corresponding IMU states was taken. An overview of all resulting quantities when compared to the respective optimized model kinematics output ($\varphi (t;{{\boldsymbol{\mathfrak{P}}}}^{* }),\dot{\varphi }(t;{{\boldsymbol{\mathfrak{P}}}}^{* })$, and $\ddot{\varphi }(t;{{\boldsymbol{\mathfrak{P}}}}^{* })$) can be found in Table 2.

## Supplementary information

Supplementary file

## Data Availability

Datasets generated during and/or analysed during the current study are available on Figshare with identifier 10.6084/m9.figshare.28271405.

## Code availability

The code used for the multi-method framework (mathematical algorithms) for sensor calibration and measurement uncertainty estimation is being made available by the corresponding author for research purposes (non-commercial use) or on reasonable request.
