# Supplementary material for: A multi-method framework for establishing an angular acceleration reference in sensor calibration and uncertainty quantification
Source: Commun Eng. 2025 Apr 7;4:65. doi: 10.1038/s44172-025-00384-8 (PMC11977018; doi:10.1038/s44172-025-00384-8)
Supplement: Supplementary file 1 — Supplementary file [file 44172_2025_384_MOESM1_ESM.pdf]

A multi-method framework for establishing an angular acceleration reference in sensor calibration and uncertainty quantification

Maximilian Gießler[1,2*], Bernd Waltersberger[1], Thomas Götz[2] and Robert Rockenfeller[2,3]

[1*]Department of Mechanical and Process Engineering, Offenburg University of Applied Sciences, Offenburg, Germany.
[2*]Mathematical Institute, University of Koblenz, Germany.
[3*]MTI Mittelrhein, University of Koblenz, Germany.

*Corresponding author(s). E-mail(s): maximilian.giessler@hs-offenburg.de;

Supplementary information

Supplementary Note 1

The scalar notation of Eqn. (8) expands to

$$\ddot{\varphi} + \mathrm{sign}(\dot{\varphi}) R_{\mathrm{g}}^{-2} \mu r_{\mathrm{b}} \left(\frac{((k_1 \ddot{\varphi} + c_1 + c_2)^2}{(\mu^2+1)^2} + \frac{(k_2 \ddot{\varphi} + c_3 + c_4)^2}{(\mu^2+1)^2} \right)^{\frac{1}{2}} + c_5 = 0 \; . \quad \text{(S1)}$$

Herein, the terms c_1 to c_4, k_1, and k_2 are defined as

$$\begin{aligned}
k_1 &= r_{\mathrm{s}_y} \cos(\varphi) + r_{\mathrm{s}_z} \sin(\varphi) - \mathrm{sign}(\dot{\varphi}) \mu \left(r_{\mathrm{s}_z} \cos(\varphi) + r_{\mathrm{s}_y} \sin(\varphi) \right) \\
k_2 &= r_{\mathrm{s}_z} \cos(\varphi) - r_{\mathrm{s}_y} \sin(\varphi) + \mathrm{sign}(\dot{\varphi}) \mu \left(r_{\mathrm{s}_y} \cos(\varphi) + r_{\mathrm{s}_z} \sin(\varphi) \right) \\
c_1 &= \left(r_{\mathrm{s}_z} \cos(\varphi) - r_{\mathrm{s}_y} \sin(\varphi) + \mathrm{sign}(\dot{\varphi}) \mu \left(r_{\mathrm{s}_y} \cos(\varphi) + r_{\mathrm{s}_z} \sin(\varphi) \right) \right) \dot{\varphi}^2 \\
c_2 &= g \left(\cos(\psi) \cos(\theta) - \mathrm{sign}(\dot{\varphi}) \mu \cos(\psi) \sin(\theta) \right) \\
c_3 &= \left(-r_{\mathrm{s}_y} \cos(\varphi) - r_{\mathrm{s}_z} \sin(\varphi) + \mathrm{sign}(\dot{\varphi}) \mu \left(r_{\mathrm{s}_z} \cos(\varphi) - r_{\mathrm{s}_y} \sin(\varphi) \right) \right) \dot{\varphi}^2 \\
c_4 &= g \left(\cos(\psi) \sin(\theta) + \mathrm{sign}(\dot{\varphi}) \mu \cos(\psi) \cos(\theta) \right) \\
c_5 &= R_{\mathrm{g}}^{-2} g (\cos(\psi) \cos(\theta) (r_{\mathrm{s}_y} \cos(\varphi) + r_{\mathrm{s}_z} \sin(\varphi)) \\
&\quad + \cos(\psi) \sin(\theta) (r_{\mathrm{s}_z} \cos(\varphi) - r_{\mathrm{s}_y} \sin(\varphi))) \; , \qquad \text{(S2)}
\end{aligned}$$

where g denotes the absolute value of the gravitational acceleration, and r_{s_y}, r_{s_z} the components of ${}^{\mathrm{P}}\boldsymbol{r}_{\mathrm{s}}$ in the y-z-plane. As the component r_{s_x} causes no torque component about the oscillation axis $\boldsymbol{e}_{\mathrm{O}_x}$, it was omitted in the further analysis. Note, Eqn. (S1) is normalized by replacing the quotient $m/I_{xx}^{(0)}$, i.e. the inverse of the squared gyradius of the pendulum around the x-axis, with the symbol R_{g}^{-2}.

Supplementary Note 2

To provide the optimizer with a suitable starting point $\mathfrak{P}_0$, several preliminary measures were taken. In particular, a reciprocal feedback loop of sensitivity analysis (Eqns. (13) and (14)) and optimization procedure (Eqn. (11)) was exploited to understand which parameters of the model were most sensitive and what parameter uncertainties were to be expected. As shown Fig. 2, the globally most sensitive parameters were R_g^{-2}, r_{s_z}, and g. Hence, for these three, we extracted the geometrical dimensions as exactly as possible from the CAD model and additionally used the precision balance `SCALTEC SBC 61` (CITRINE Solutions LTD., Oranit, West Bank) of accuracy class II to determine the mass up to 0.1 g, yielding $R_g^{-2} = 1.94\,\mathrm{kg} / 0.246\,\mathrm{kg\,m^2} = 7.88\,\mathrm{m^{-2}}$ and $r_{\mathrm{s}_z} = 0.339\,\mathrm{m}$. From this procedure, we also obtained $r_{\mathrm{s}_y} = 0.00005\,\mathrm{m}$. Note that (i) the mass of the pendulum also includes the moving part of the bearing and (ii) the mass distribution of the pendulum was designed to minimize $|r_{s_x}|$ to prevent asymmetric bearing load with respect to the spatially separated roller bearings. For the gravitational constant, the "g-Extractor"

provided by the German "Physikalisch-Technische Bundesanstalt" (Physical-Technical Federal Institute, www.ptb.de) yielded a value of $g = 9.8087\,\mathrm{m/s^2}$ for the location (latitude and height above sea level) of our lab. Initial values φ_0, $\dot{\varphi}_0$ for the model ODE (Eqn. (3)), as well as the offset ${}^{\mathrm{M}}\boldsymbol{r}_0$, were taken from the MOCAP system. In particular, for experiment E1 $\varphi_0 = -1.0234\,\mathrm{rad}$, $\dot{\varphi}_0 = 0.0053\,\mathrm{rad/s}$ and for experiment E2 $\varphi_0 = -0.4386\,\mathrm{rad}$, $\dot{\varphi}_0 = 0.0298\,\mathrm{rad/s}$, while ${}^{\mathrm{M}}\boldsymbol{r}_0 = (0.91\,\mathrm{m}, -1.40\,\mathrm{m}, 1.47\,\mathrm{m})^T$ in both cases. Transformation angles $\beta = 0.0070\,\mathrm{rad}$, $\gamma = -0.0054\,\mathrm{rad}$, $\theta = 0.0014\,\mathrm{rad}$, and $\psi = 0.0017\,\mathrm{rad}$ were chosen as the result of the preliminary optimization when starting from zero. For the lever arm of applied friction force, r_b, we took the approximate outer diameter of the ball contact points from the ball bearing data sheet, where it was given as 9.6 mm. For the friction coefficient, no data sheet was available and the initial value was chosen based on an experienced guess as $\mu = 0.02$ for experiment E1 and $\mu = 0.06$ for experiment E2. Finally, the body-fixed positions of the markers with respect to the pendulum were taken from the CAD model as ${}^{\mathrm{P}}\boldsymbol{r}_1 = (0, 0, -0.1)^T$, ${}^{\mathrm{P}}\boldsymbol{r}_2 = (0, 0, -0.2)^T$, ${}^{\mathrm{P}}\boldsymbol{r}_3 = (0, 0, -0.3)^T$, and ${}^{\mathrm{P}}\boldsymbol{r}_4 = (0, 0, -0.4)^T$ all measured in meters.
